# A review of microscopic cell imaging and neural network recognition for synergistic cyanobacteria identification and enumeration

**DOI:** 10.1007/s44211-021-00013-2

**Published:** 2022-02-25

**Authors:** Liam Vaughan, Arash Zamyadi, Suraj Ajjampur, Husein Almutaram, Stefano Freguia

**Affiliations:** 1 Water Research Australia, Level 2, 250 Victoria Square, Adelaide, SA 5000 Australia; 2 Department of Chemical Engineering, The University of Melbourne, Parkville, VIC 3010 Australia; 3 Department of Civil and Mineral Engineering, University of Toronto, Toronto, ON M5S 1A4 Canada

**Keywords:** Cyanobacteria, Imaging microscopy, Cytometry, Machine learning, Quantitative phase imaging, Cell recognition, Workflow

## Abstract

Real-time cyanobacteria/algal monitoring is a valuable tool for early detection of harmful algal blooms, water treatment efficacy evaluation, and assists tailored water quality risk assessments by considering taxonomy and cell counts. This review evaluates and proposes a synergistic approach using neural network image recognition and microscopic imaging devices by first evaluating published literature for both imaging microscopes and image recognition. Quantitative phase imaging was considered the most promising of the investigated imaging techniques due to the provision of enhanced information relative to alternatives. This information provides significant value to image recognition neural networks, such as the convolutional neural networks discussed within this review. Considering published literature, a cyanobacteria monitoring system and corresponding image processing workflow using in situ sample collection buoys and on-shore sample processing was proposed. This system can be implemented using commercially available equipment to facilitate accurate, real-time water quality monitoring.

**Graphical abstract:**

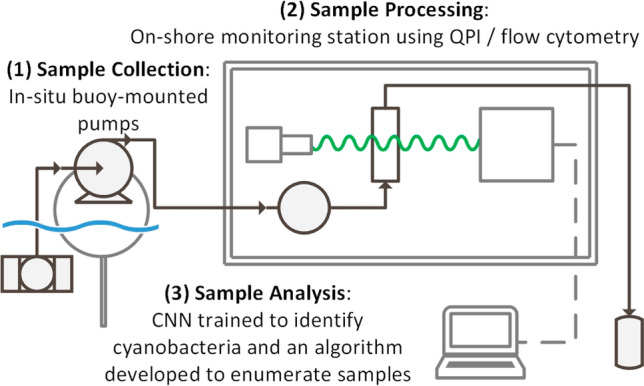

## Introduction

### Consequences of cyanobacteria and harmful algal blooms

Proliferation of harmful algal blooms (HABs) consisting of microorganisms such as potentially toxic cyanobacteria has becoming an increasingly common occurrence within freshwater systems globally [1]. Anthropogenic activity and climate change has likely exacerbated HAB prevalence due to increases in significant cyanobacteria biomass drivers, including increased nutrient uptake due to urban and agricultural activity, increased water temperature, and increased mean water residence time [2]. Risks to human populations arise since water reservoirs supplying potable water often provide environments suitable for microbial colonisation. Cyanobacteria can also accumulate within a water treatment plant due to a low influx of cyanobacteria cells, introducing risks to water quality and safety despite the absence of a HAB at the water source [3]. Growth of such microorganisms poses significant health, social, environmental, and economic implications.

Social impacts result from the production of unpleasant aromatic compounds during microbial growth and decay, imparting undesirable odours and flavours in the water. These “off-flavours” occur due to both benign and harmful cyanobacteria, so the growth of any cyanobacteria must be carefully monitored. The presence of even miniscule concentrations of cyanobacteria in water may thus lead to customer dissatisfaction and loss of confidence with water supplies [4].

Many cyanobacteria species found within HABs release cyanotoxins that cause several negative human and animal health effects, including respiratory and allergic reactions, gastritis, liver damage, and neurotoxicity [5]. Long term exposure is associated with increased tumour growth, while acute cyanotoxin exposure may be fatal. Exposure to waters containing cyanobacterial cells in quantities approaching or exceeding 100,000 cells/mL is associated with a moderate probability of adverse health consequences. [6]

HABs also have considerable effects on the stability and health of water ecosystems. Cyanobacterial growth reduces the irradiance of solar energy through water columns, reducing the energy accessible to other organisms within the ecosystem. Cyanobacteria also consume free CO_2_ within water bodies, restricting the access of other plant species. This interrupts the production of oxygen within the ecosystem, causing rapid hypoxia and death for aquatic animals. Many of the cyanotoxins produced by cyanobacteria that affect humans also harm ecological systems [7].

Economic costs arise as secondary consequences of HABs through reductions in value of affected water bodies and costs incurred in both reducing bloom risks and treating water. The health impacts caused by cyanotoxins negatively affects the usability of water for drinking and in agriculture, while unacceptable tastes and odours further diminish water usability. HABs can also eliminate recreational usability of water bodies, potentially impacting local tourist industries. Recurring HABs will cause long-term declines in tourist activities [8]. A report prepared for the Land and Water Resources Research and Development Corporation in 2000 estimated total annual costs of HABs in Australia as 180–240 million AUD [9]. Climate change is projected to continually increase this economic cost due to increased water temperatures and climatic impacts on nutrient transport driven by hydrology [10].

### Water quality microscopy and cell imaging

Inspecting the species within a water sample at a microscopic level provides detailed information regarding the taxonomy, quantity, and viability of microorganisms present within the sample. Continuously monitoring populations of species such as cyanobacteria can assist the early detection of HABs, determine the efficacy of water treatment methods used, and allow specific risk assessments by considering the taxonomy of species present and their associated cyanotoxins, as well as the hazard posed at specific cell densities.

Cell viability analyses are used to assess the risk posed by microorganisms on water quality as they determine the residual risk posed by water following treatment processes. Water treatment plants aim to improve water quality through the elimination of hazardous contaminants, including waterborne microbial populations. This is achieved through several possible treatment methods, including ozonation, chlorination, ultraviolet radiation, activated carbon filtration, and clarification through coagulation and sedimentation [11, 12]. The efficacy of these treatment methods for the reduction of microbial activity can then be measured through cell viability assessments. These assessments traditionally require determination of cell membrane integrity using a combination of DNA-binding labelling agents, such as cell-permanent nucleic-acid dyes for living cells and cell-impermanent nucleic-acid dyes for dead cells [13]. Emerging label-free viability assessments using imaging microscopy offers simplified non-destructive analyses [14]. The potential for the techniques considered within the literature to assist cell viability analyses is discussed within the respective subsections and summarised within Table 1.

**Table 1:** Microscopic imaging technology summary

Category	Name/technique	Advantages	Disadvantages
Imaging flow cytometry	FlowCam 8000 series	Higher maximum magnification (×200) Colour imaging Organism parameters automatically measured Automatic cyanobacteria detection using fluorescence microscopy Wide temperature operation range (4–40 °C)	Low sample processing rate (0.05 mL/min) Minimum sample size 2 μm (too small for some species) Limited in situ applications No inbuilt cell viability assessment capabilities (no dye injection)
	Amnis ImageStream MkII Imaging Flow Cytometer	Higher sample processing rate (0.25 mL/min, 5000 cells/second) Enhanced data collection (12 images/cell at different angles) Automatic cyanobacteria detection using fluorescence microscopy	Lower maximum magnification (20×/40×/60×) Limited in situ applications Minimum sample size approximately 5–7 μm (too small for some species) No inbuilt cell viability assessment capabilities (no dye injection)
Phase-contrast	Quantitative Phase Imaging (QPI)	Capable of assessing cell viability/capturing images without labelling agents Non-destructive for samples Enhanced biophysical data availability (dry-mass, internal structures) Proven integration with neural networks 3D cell images can be digitally reconstructed	Limited commercial availability of QPI devices Limited in situ applications Greyscale images Lower sample throughput compared to imaging cytometry
Emerging	Helium microscopy	Does not disturb sample during imaging Provides surface chemical composition data	Difficult imaging conditions (Sample must be in a vacuum) Minimum sample size approximately 50 μm Images take hours-days to produce per sample
	Scanning electron microscopy	Very precise, high-resolution images	Images are unnecessarily detailed Expensive Equipment is limited to laboratory applications Samples must be coated with conductive material

Traditional water quality microscopy typically involves cultivating microorganisms from a water sample and then fixing a sample of the microorganism on a slide, which is analysed under a brightfield microscope. This method is less effective than more modern techniques discussed within this review, as it requires a lengthy and labour-intensive sample preparation process and introduces experimental errors. [15].

Consideration of the advantages, disadvantages, and uses of these techniques within the published scientific literature, and specifications of available technologies indicates the limitations and practical applications of these techniques within the context of water quality analysis and neural network recognition. This informs the potential applicability of these techniques to identify and enumerate cyanobacteria within a water sample, expanded further within the recommendations section of this review.

### Machine learning and cell recognition

Presence of cyanobacteria within a sample can be ascertained through fluorescence microscopy, which considers the intensity of light scattered by pigments such as chlorophyll-*a* and phycocyanin within cells to determine cyanobacteria cell density and thus attain an approximate cell count [16]. This technique is useful for determining presence of cyanobacteria with a sample, however provides limited information about specific cyanobacteria species or quantities, requiring further microscopic analysis for more detailed taxonomic identification and enumeration.

Identification and enumeration of imaged cells using conventional sample preparation and brightfield microscopy allows direct analysis of a cyanobacterial bloom, however this manual method is labour intensive and requires significant technical and morphological expertise. [17].

The literature review of recent advances in artificial intelligence (AI) image recognition using machine learning-trained neural networks determined that cell recognition using neural networks presents a viable alternative solution for specimen identification using information provided by microscopic imaging technology. Accurate and rapid classification of cyanobacteria using such images continues to present a challenge for machine learning algorithms due to microscopic but diverse cell sizes, polymorphism, and lack of taxonomically revealing sexual reproductive structures in cyanobacterial cells [18]. Image recognition algorithms consider information and spatial parameters contained within a given image to identify a subject. Increasing the number of measured cellular parameters allows a higher dimensional (the neural network increases in complexity) data analysis, improving recognition capabilities and providing higher-confidence statistical results [19, 20]. Imaging methods with greater information uptake thus have greater potential to assist AI recognition, increasing the value of methods that provide more data to neural networks.

### Proposed water quality monitoring system

Recommendations constructed following the investigation of published literature considered three potentially promising quantitative phase imaging (QPI) methods—a portable QPI unit (Method 1), QPI using Michelson interferometry (Method 2), and a commercially available in situ probe (Method 3). Table 1 compares the value of these three methods to facilitate accurate cell recognition, with Method 2 considered the most viable approach. A proposed cyanobacteria monitoring system and image processing workflow using sample collection buoys and Method 2 is then outlined in Fig. 4 and discussed further.

## Microscopic imaging technology

This section constitutes a literature and technological review of commercially available and emerging microscopic imaging techniques, and the practical water quality microscopy applications of these techniques. Table 1 outlines a summary of the advantages and disadvantages of the techniques discussed in greater depth within this section.

### Flow cytometry

Flow cytometry involves the inspection of a cell culture within a flowing sample solution (the sheath fluid) using optical and/or fluorescence imaging devices. The primary advantage of this technique is that it offers a considerably less labour intensive, less time-consuming, and more accurate water quality analysis method relative to conventional techniques using brightfield microscopy and heterotrophic plate counts [13, 21, 22].

Methods which require sample incubation or preservation introduce inaccuracies, as these conditions may alter sample composition immediately or over time. Different nutrient agars may inhibit certain microorganisms preferentially, introducing risks of substrate inhibition causing species within water to remain undetected after later sample analysis [15]. Enumerations of cyanobacteria populations within preserved and live samples using FlowCam found higher cell counts in preserved samples due to post-collection cell multiplication [18], indicating that inaccuracies can by mitigated through real-time in situ live sample analysis.

Standard cultivation and analysis methods require specialised training and manual analysis by technicians, which takes 1–3 days for bacterial samples and up to weeks of incubation for viral samples [13, 15]. High sample processing times are particularly problematic in water treatment contexts, where treated water is often directly supplied to consumers. Contaminated water may be distributed before hazards within the water are identified, introducing health risks to consumers [13].

#### Non-imaging flow cytometry

Traditional non-imaging flow cytometry involves the analysis of suspended particles within a flowing sheath fluid such as bacteria, viruses, protozoa, cell fragments, and inorganic debris based on how these particles scatter light or fluoresce when travelling through a laser beam. The forward scatter signal (FSC) is relative to particle size, while side scatter signals provide insight to sample complexity and granularity [13, 22]. The non-imaging method introduces many limitations to the accuracy of cell population and morphology analysis. One such limitation is that light scattering is not an absolute quantity but is instead relative, with factors such as the sample’s surface roughness, refractive index, and the sheath fluid used found to influence the magnitude of light scattered. A cell with a FSC larger than a neighbouring cell by a factor of two will thus not necessarily have a size double that of the neighbouring cell, complicating taxonomic identification efforts. Scatter signals are also affected by the particle’s orientation at the point of analysis if the particle is of an irregular shape, while clumped cell clusters may be registered as a single larger particle. Cells with smaller diameters weakly and inconsistently scatter light, while larger cells preferentially scatter in the forward direction, posing potential accuracy limitations for detection of cells and analysis of populations [13].

#### Imaging flow cytometry

Imaging flow cytometry offers an alternative approach to cell population and morphological analysis by capturing real-time images of cells in a flowing sheath fluid, which can then be provided to an operator or AI algorithm for further enumeration and taxonomic identification. As previously discussed, imaging cell populations using flow cytometry provides significantly expedited analysis with less manual preparation required [13, 15].

Leading commercially available imaging flow cytometry systems include the FlowCam (8000 Series, Yokogawa Fluid Imaging Technologies) and Amnis ImageStream (MkII, Luminex). The advantages and disadvantages of these systems are discussed in Table 1, with relevant system specifications outlined in Table 2.

**Table 2:** Imaging flow cytometer specification comparison [23, 24]

	FlowCam—8000 Series	Amnis ImageStream MkII
Min. particle size	2 µm	7 µm
Max. magnification	×200 objective magnification (50 µm field of view)	×60 objective magnification (40 µm field of view)
Sample processing capability	0.05 mL/min at ×200 magnification	0.25 mL/min at ×60 magnification
Camera	Monochrome or colour conductive metal oxide sensor (CMOS)	Colour charge coupled device (CCD)
Fluidics	“Micro syringe pump” to optimise flowrate	“Sheath fluid syringe pump”
Physical characteristics	36 W × 38H × 44D (cm) 23 kg	89 W × 66H × 63.5D (cm) 182 kg
Operational requirements	100–240 V AC power	100–240 V AC power
Dye injection capabilities	Not included	Not included
Sterilisation	Self-cleaning	Self-cleaning

Algal and cyanobacterial growth may range from benign to hazardous, depending on the species present. Risk assessments that directly consider the microbial taxonomy, particularly in terms of potentially toxic cyanobacteria, of species in a water sample thus increases the accuracy of risk assessment processes for humans, agriculture, and environmental systems [5, 18].

Cell viability assessment methods utilising automated imaging flow cytometry systems also provide simplified and expedited assessments when compared with standard techniques, such as manual counting using a hemocytometer [25]. Using imaging cytometry, target cells are stained with one or more dyes to achieve optimal cell visibility (discussed further in *Sample processing—OC-300*). A combination of fluorescence and brightfield images of stained cells can provide total, dead, and live cell counts [25]. Viability is determined as the proportion of live cells to total cells, providing an indication of the performance of a water treatment process and the residual risk posed by the treated water sample [11]. Table 2 outlines the automated dye injection capabilities of two commercially available imaging flow cytometers, which indicates the ability for these cytometers to facilitate cell viability assessments.

Imaging flow cytometry systems aim to maximise cell imaging rates, while also maximising the resolution of images captured. Modern camera technology widely used in imaging flow cytometry techniques introduce fundamental trade-offs which must be considered to determine a technique’s feasibility [19]. Increasing fluid velocity and imaging speed results in the camera collecting fewer photons for each specimen, leading to a reduction in sensitivity. Sample throughput can be increased while maintaining low fluid velocities and imaging speeds using imaging flow cells with many parallel channels. McKenna et al. [26] demonstrated high-throughput imaging flow cytometry using flow cells with 384 parallel channels, however this technique requires a large field of view which necessitated an imaging lens with a lower numerical aperture and thus compromised the spatial resolution of the images captured [19, 26]. Resultantly, a flow cytometry technique with a higher sample throughput rate or higher imaging resolution is not objectively superior to alternatives, with its practicality determined by its required application. Applications such as neural network recognition of microscopic cells rely on superior information and must prioritise spatial resolution to achieve high recognition accuracy [17, 20, 27].

Researchers used FlowCam to enumerate and taxonomically identify cyanobacteria species present within both early and advanced-stage HABs across tens of freshwater systems in Alberta, Canada. Utilising FlowCam at its maximum possible objective magnification (× 20 for the model used by researchers) yielded the highest total cell counts, exceeding those yielded by a × 10 objective by a factor of four, and identified two to four times more cyanobacteria species using increased magnification [18]. This finding agrees with those of similar taxonomic investigations, which found higher magnification necessary for accurate species-level identification [28, 29].

Attempts to classify FlowCam images using trained neural networks have considered larger species including *Oscillatoria *sp. and *Anabaena *sp. or entire colonies of *Microcystis *sp. due to the lack of information available in images captured of smaller species [30]. QPI presents a potential solution as it provides images with additional quantified biophysical parameters, improving information availability and image recognition accuracy.

#### Sample preparation—OC-300

OC-300 is a commercially available automation unit developed by OnCyt Microbiology. The unit is designed to act as an intermediate between raw samples collected from water bodies and flow cytometry equipment, with acquired water samples manually or automatically provided to OC-300. Up to 12 parallel sample streams can be prepared by OC-300, allowing a single flow cytometry setup to continuously measure cell counts from multiple locations. The device’s autoloaders prepare a sample to a desired volume between 100 and 500 µL, which can also be automatically labelled by a dye to facilitate easier sample analysis. [31].

Researchers studying cyanobacteria populations to track early and advanced-stage HABs in Alberta, Canada using FlowCam encountered issues with bacterial cells clogging smaller imaging flow cells [18]. Addition of Lugol’s solution was found to reduce cell adhesion, improving image capture efficiency as cells were more evenly dispersed across the flow cell. While adding Lugol’s solution prevented chlorophyll-*a* autofluorescence detection [18], this poses fewer limitations on a system that measures cyanobacteria presence using image recognition via machine learning. If cell counts exceed a threshold at which clogging is likely to occur within imaging cells, the OC-300 may also dilute the solution to allow easier sample processing [31].

The precisely prepared samples can then be injected into an adjacent imaging flow cytometer such as the devices outlined in Table 2 or used to prepare samples for analysis by QPI. Synergy between these devices allows precise sample preparation, providing accurate cell counts, providing accurate viability assessments, and facilitating taxonomic identification of the species present. Proposed integration of OC-300 into a HAB monitoring station is further discussed in the recommendations section.

### Quantitative phase imaging

QPI, also known as holographic microscopy, is a phase-contrast microscopy method useful in non-intrusive cell morphological studies. QPI detects cells based on the scattering of certain wavelengths of light as they pass through a sample, which creates a contrast against the background where the light is unobstructed and does not scatter. Examined samples create contrast proportional to their thickness and internal refractive index inhomogeneity, which determines the total amount of light scattered and provides information about the internal and external biophysical properties of the cell. This permits precise analysis of internal and external cell parameters including density, morphology, mass, volume, membrane, and cell structures including organelles [32, 34].

Integration of QPI units with flow cytometry equipment provides capabilities for enhanced analysis relative to conventional imaging flow cytometry techniques (Table 2). Combined flow cytometry QPI devices process samples at rates considerably higher than those achievable through manual analysis, expediating specimen identification and providing larger data sets for neural network training [20, 35, 37]. In addition to capturing high resolution images, additional biophysical parameters not accessible through conventional imaging flow cytometry are made available, including those previously outlined [37]. These parameters provide valuable assistance in taxonomic identification efforts, and the quantitative nature of the data obtained through QPI is particularly useful for machine learning applications [32]. Synergistic cell recognition using QPI data and trained neural networks is discussed further later in this review.

Tomographic QPI techniques can be used to construct a 3D refractive index tomogram of the specimen, revealing further spatial information regarding a specimen’s internal structure. Multiple 2D QPI images combined with a reconstruction neural network provides a tomographic image, assisting taxonomic identification efforts [34, 38]. Constructing 3D tomograms for each cell analysed is however a computationally intensive process, since the tomographic reconstruction algorithm must be applied at many depths of focus to digitally capture the cell properties across its volume [39].

Many QPI techniques have been used within different research contexts, including off-axis Fresnel holography, Fourier holography, image plane holography, Gabor holography, in-line holography, and phase-shifting digital holography [34, 40]. Many of these techniques are of limited use in biological analysis as they create twin images, which reduce pixel array use efficiency and thus limit the information captured in each image. In-line and phase-shifting holography are valuable in biological analyses, as these techniques suppress twin images and thus utilise entire hologram pixel counts [40].

Phase-shifting QPI is a common method for detailed microscopic analyses of samples, and uses a combination of mirrors, beam-splitters, and lenses to create an interference pattern on an imaging surface [40]. Examples of this technique include Methods 1 and 2, discussed in the “Recommendations” section.

In-line QPI is an alternative technique used in many in situ contexts due to the simplicity of the optical components required for this configuration. For samples analysed using in-line QPI, illumination is provided by a point source such as the end of a fibre-optic cable. The light waves emitted from this source propagate through the sample solution and scatter off any objects within the region of analysis. The interference pattern produced when scattered waves interfere with the primary wave is recorded by a charge-coupled device (CCD). A wave reconstruction algorithm is then applied to retrieve an image of the objects examined [34, 41]. Portable probes (such as Method 3) using in-line QPI are a valuable emerging in situ analysis technique.

#### Applications of QPI

The enhanced specimen information collection capabilities and high-resolution 3D imaging potential provided using QPI has driven this technique’s use within a range of scientific and biomedical contexts.

##### Biomedical applications

QPI analysis of biological samples has been particularly attractive within medical contexts due to the detailed cell-specific 3D images attainable using this method, providing valuable diagnostic capabilities. Red blood cell analysis using QPI has allowed the detection and identification of leukemic red blood cells, and the real-time monitoring of T-cells terminating cancer cells [42]. Morphologies of red blood cells, particularly cell membrane deformability, within blood samples screened using QPI data have allowed the identification of diabetic blood cells [43]. 3D tomographic images were produced during a study of red blood cells parasitised by malaria-inducing *Plasmodium falciparum*, providing fine detail on subcellular structures of the host blood cells and invading parasite, with structural and chemical characteristics recorded during stages of parasite maturation [44]. A similar approach to that used by Lee et al. [43] may be used in water quality analyses to diagnose the presence of specific cyanobacteria species, such as toxic varieties, within a particular water system. The monitoring of the cell viability conducted by Popescu [42] presents a method for determining the effectiveness of treatment methods by analysing cell integrity before and after treatment processes.

##### Portable QPIU

A portable quantitative phase imaging unit (QPIU) was used by Jo et al. [20] for the synergistic imaging and recognition of *Bacillus anthracis* within a sample containing unlabelled living cells. The neural network trained and used by Jo et al. [20] is discussed in greater depth later in this review. A further analysis of the advantages and disadvantages of this method is discussed in the recommendations section (Method 1).

This technique was used by researchers to obtain quantitative phase images of 1 µm bacteria including *B. anthraxis, B. cereus,* and *B. subtilis*. This QPI data were used to train a CNN “HoloConvNet” for up to 96.3% recognition accuracy. [20].

This portable QPIU was developed with the requirement that it could be easily transported to and from a BSL-3 facility at the South Korean Agency for Defense Development [20]. It consists of a charge coupled device (CCD) camera mounted at the exit of a tube containing two linear polarisers and a Rochon prism. Monochromatic 532 nm laser light is passed through a sample placed within an imaging cell, which enters the objective of a standard optical microscope at 100 × magnification under oil immersion [20]. Refractive index information is encoded within the spectrum of light as it passes through the sample [32]. The first polariser linearly polarises the light, which is equally divided into two beams with slightly different propagation angles by the Rochon prism. The two beams become parallel after passing through the second linear polariser. An interference pattern is then created on the surface of the CCD camera, with the position of the linear polarisers adjusted to produce an optically focussed image. A quantitative phase image is then retrieved from the information using a standard field retrieval algorithm. [20].

Further research may investigate potential optimisations of this design for water quality analysis, such as the integration of this QPIU with flow cytometry equipment for improved analysis automation.

##### Michelson interferometry

**Fig. 1:**
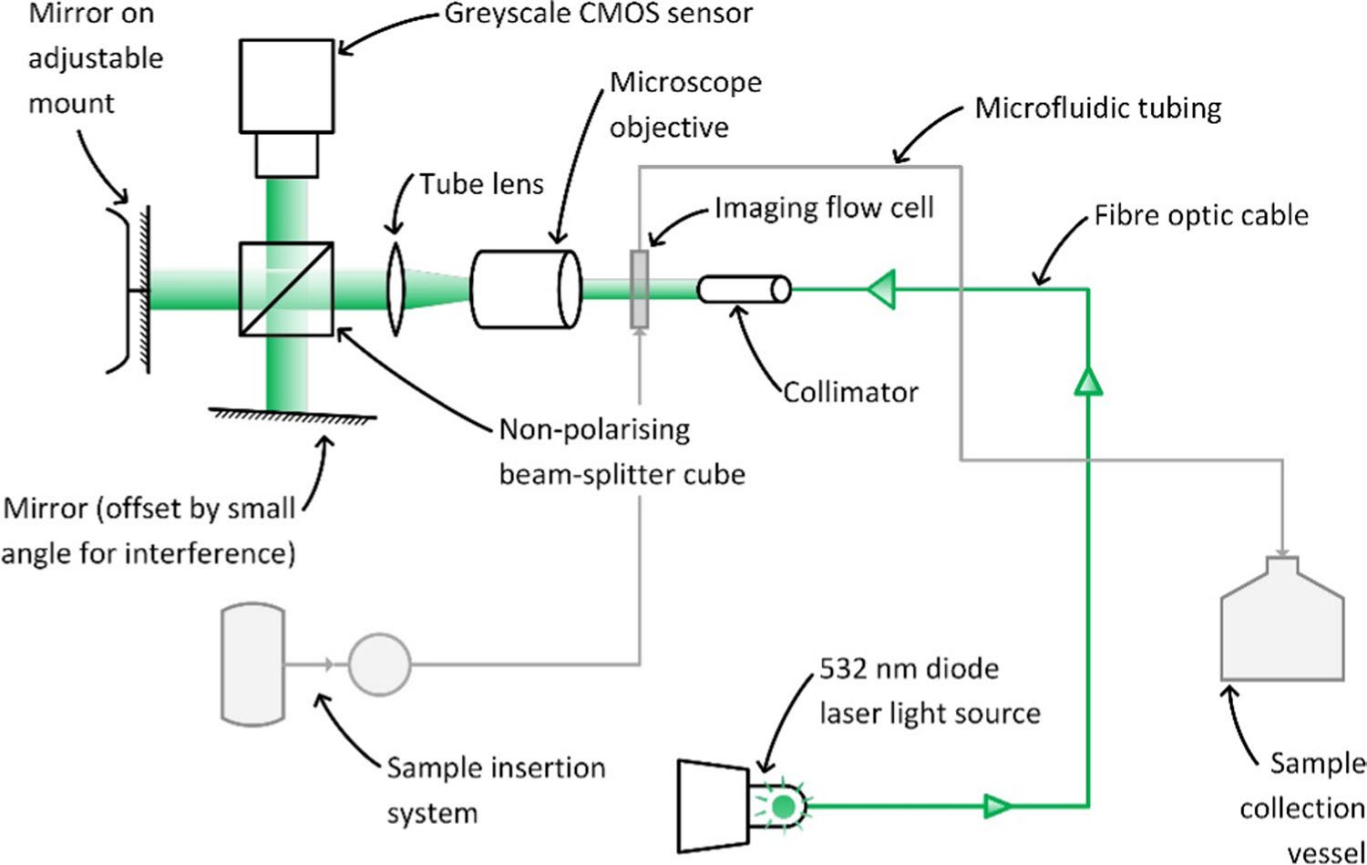
Schematic diagram of QPI using Michelson interferometry. Figure [37] adapted from Min et al.

**Fig. 2:**
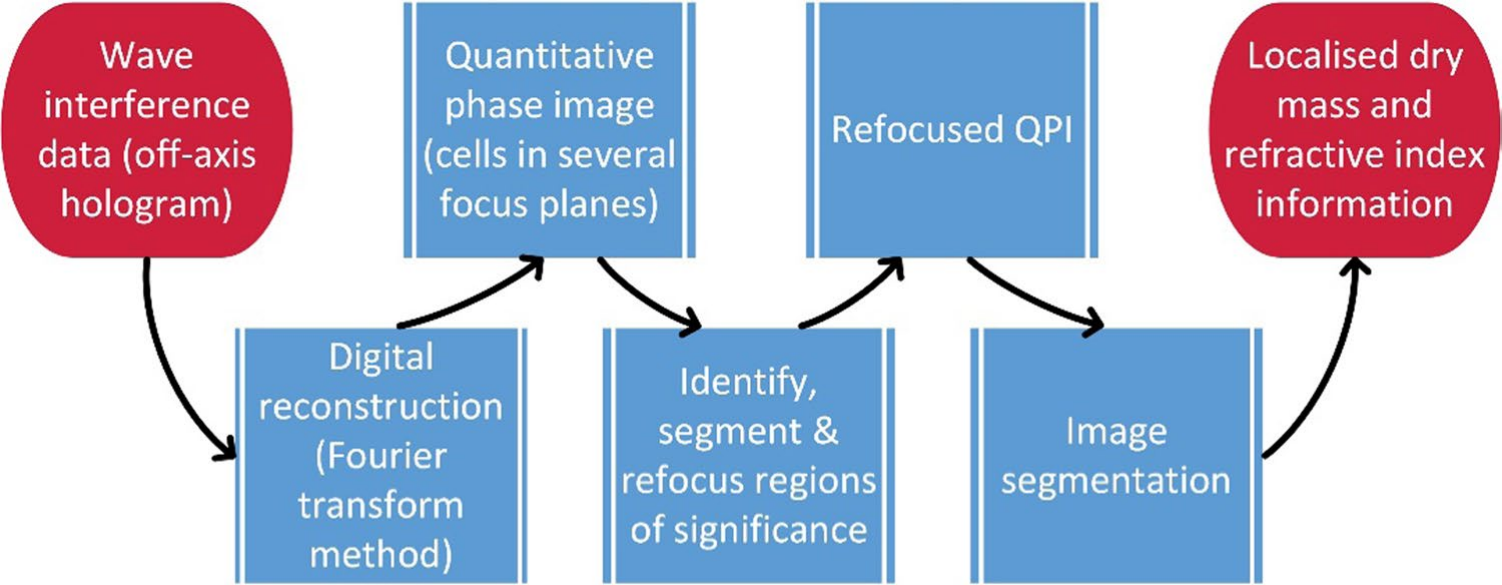
Image post-processing workflow used by Min et al. [37]

Quantitative phase images were captured of tumour cells using Michelson interferometry (Fig. 1), allowing precise retrieval of biophysical cellular features including radius and dry mass. Integrated flow cytometry was used to transport sample solution through an imaging flow cell, providing automated sample processing and data collection [37]. Captured images were then processed as specified in Fig. 2. The advantages and disadvantages of this method are further considered within the “Recommendations” section (Method 2).

Min et al. [37] developed a QPIU that utilises phase-shifting quantitative phase imaging, with an interference pattern created on the lens of a complementary metal–oxide–semiconductor (CMOS) camera by Michelson interferometry. As with the portable QPIU (Method 1), a 532 nm monochromatic laser is used as the illumination source. The laser beam is directed through an imaging flow cell arranged perpendicular to the direction of beam propagation. Cells within the sample solution pumped through the flow cell pass through this beam, causing phase shifts due to their refractive indexes. A microscope objective and tube lens magnify the beam, which is split into two identical beams by a non-polarising beam-splitter cube. These beams are then reflected by two mirrors at slightly different angles, creating an interference pattern when the beams are recombined. This interference pattern is focussed on the surface of the camera, which continuously captures images at 100 frames/second. Quantitative phase information is then retrieved from the images using a reconstruction algorithm such as the Fourier transformation method. The image processing method used by Min et al. is outlined in Fig. 2 [37].

As indicated above, precise biophysical cellular properties were measured by Min et al. [37], which demonstrates significant value to a cell detection neural network. Radii of examined tumour cells in flow were measured with uncertainties of only 50–60 nm, while dry mass values were determined with uncertainties of 4–6 picograms. The measured dry mass and radius values for cells in flow aligned closely with those attained of static cells, indicating the reliability of this method and that flow cytometry analyses are not associated with a significant loss of accuracy. [37].

##### WaterScope

QPI technology has been used to develop WaterScope, which has been used by researchers to image microscopic flora and fauna in Lake Balaton, Hungary [17, 39]. A lens-free prototype setup was used to image microorganisms within the 10–200 µm size range, with researchers estimating this method to inspect samples 50–100 times faster than a similar analysis conducted with conventional microscopy. Unlike most QPI devices, WaterScope is capable of capturing colour images of the examined specimens by using three different coloured lasers as the incident light. The researchers prioritised colour imaging as a means of simplifying taxonomic identification. A simple pattern recognition algorithm was then trained by researchers and subsequently used to identify imaged microorganisms with 90–95% accuracy [39]. WaterScope, while not commercially available at the time of writing, indicates the viability and value of QPI within water quality contexts to facilitate automated identification and enumeration of samples.

##### 4Deep S7 submersible microscope

The S6 submersible microscope presents as an early example of commercially available QPI designed for in situ water quality analysis [34]. Researchers have used this submersible holographic microscope to image and identify aquatic microorganisms, capable of operating within environmentally hazardous regions including the high Artic Ocean [41].

This device uses digital in-line holographic microscopy, allowing simplified image collection without requirements for polarisers and mirrors. The design of the device utilises a gap between the light source and the camera through which natural convection transports the water. This allows the device to capture images of organisms within the water as they passively pass through the device, allowing a qualitative analysis of the taxonomy of the sample and an approximation of cell counts. This passive monitoring approach however prohibits a quantitative analysis (i.e., no accurate cell counts can be attained) as the volume of water passing through is not precisely known [45].

#### Advantages of QPI

The primary advantage of QPI as a cell analysis technique is the increased information provided by this technique relative to brightfield microscopy and imaging cytometry. As previously discussed, endogenous refractive index can be correlated to the structural and biochemical characteristics of each target specimen [20]. This refractive index data also allows the determination of dry-mass and volume at a single cell level since refractive index is linearly proportional to cell density, providing invaluable information to assist in the identification of cells using a neural network [32].

An additional significant advantage of QPI analysis compared with imaging and non-imaging cytometry is the label-free analysis technique used. Labels such as cell viability indicators, fluorescence markers, and contrasting agents may affect the physical properties of a sample, reducing analysis accuracy. Such labels are not required in QPI, providing a more objective morphological method [32].

A diverse range of specimen sizes can be analysed using QPI since quantitative phase data can be obtained from both wave amplitude and phase information. Larger specimens will scatter or absorb more incident light and thus lower the amplitude of outgoing light [46], rendering them easily visible against the background light. Smaller specimens including single celled organisms may not absorb or scatter significant amounts of light in the visible spectrum, rendering them difficult to detect using brightfield microscopy. These small specimens are called phase objects as they do not significantly affect the amplitude of light passing through them but instead cause a phase shift. The phase interference method used in QPI thus allows enhanced analysis of phase objects and increases the range of organisms that can be examined without labelling agents [32].

#### Disadvantages of QPI

QPI offers significantly more target cell information relative to brightfield microscopy, however captured images are typically monochromatic. While recognition algorithms can utilise cellular information such as colour to classify a species [47], the provision of localised dry-mass information using QPI is more valuable as it provides the algorithm information about internal and external cellular structures [32].

Reduced maximum sample throughput rates attainable using QPI is a significant limitation of this microscopic imaging method. When compared with throughputs attainable using non-imaging flow cytometers (order of 100,000 cells/second), QPI offers a maximum rate of analysis two orders of magnitude lower [36]. Like the fundamental compromise between speed and sensitivity induced by the cameras used in imaging flow cytometers, QPI imaging speeds are also limited by camera technology. Increasing sample throughputs thus decreases image resolution and can induce motion blur, with subcellular image resolutions often sacrificed to improve sample throughput rates. At high throughputs, image field of views are limited to between tens and hundreds of cells. The resolution of these images must be reduced to compensate [36]. A combination of emerging ultra-high throughput QPI techniques and deblurring algorithms discussed further in this review present a potential solution to this limitation.

#### Emerging QPI techniques

As a technology with many emerging applications, QPI continues to be the subject of ongoing research and improvement. Synthetic aperture approaches aim to extend maximum spatial resolution by a factor of two, allowing a greater amount of information to be resolved from a single specimen. Additional specimen information can also be resolved by combining fluorescence microscopy with QPI, the additional data from which improves machine learning capabilities and recognition accuracies [32].

Lee et al. [36] aimed to overcome the maximum throughput limitations of contemporary QPI technology through the development of multiplexed asymmetric-detection time-stretch optical microscopy (Multi-ATOM). This technique permits ultra-large-scale classification of single cell samples using integrated QPI and flow cytometry, with maximum sample throughput rates and analysing capabilities exceeding that of classical QPI by a factor of 100 [36]. Researchers achieved sheath fluid flowrates of up to 2.3 m/s, with images captured at a rate of 10,000 cells/s. The phase gradient of light passing through each cell was measured, with a knife edge used for partial beam blockage. This allowed the measurement of intensity variation across the dimensions of the cell which provided a detailed image. Phase and amplitude contrast data were also used to reconstruct greyscale brightfield contrast images of each cell [36].

The time-stretch technique used in this method is an emerging optical imaging concept. The combination of a light beam splitter and a dispersive medium (such as a fibreoptic cable tens of kilometres long) allows the encoding of spatial cell information into the spectrum of a pulse of broad-spectrum light. Time-stretch images are captured at frame rates with a megahertz order of magnitude, compared with the order of 100 frames per second attainable with classical techniques [19]. This real time imaging approach is ideal for large scale imaging of individual cells, providing a method for greatly increasing data collection rates.

### Emerging/developmental technologies

#### Scanning electron microscopy

Recent efforts have seen the use low-voltage scanning electron microscopes (SEM) in flow cytometry. This research aimed to use a SEM for the characterisation of objects in platelet concentrate storage, which required the use of highly sensitive equipment exceeding the capabilities of conventional optical microscopy to detect objects with dimensions ranging from 25 to 700 nm. Conventional SEMs require samples to be coated with a layer of conductive metal with a thickness comparable to that of small extracellular vesicles, however low-voltage SEM approaches used by researchers obviated the need for this layer, enabling the examination of objects smaller than single cells [48, 49].

The main advantage of SEM technology is the high resolutions of images captured, which provides valuable and detailed information to a machine learning algorithm used to classify the images. When combined with other data collected from examined microorganisms, including dry-mass and fluorescence readings, greatly enhanced recognition capabilities and accuracies can be attained.

Despite the enhanced resolution of SEM images, this technology is unlikely to be applied to large scale water quality microscopy due to a few significant disadvantages. Individual SEM units are large, expensive, and must also be operated and stored in areas free from vibrational, electric, or magnetic interference. They must also be supplied with a consistent voltage to facilitate circulation of cooling water and require a skilled and specially trained operator. These requirements prohibit the use of SEM for any in situ purposes due to risks of equipment damage, significantly impacting the applicability of this technology for water quality monitoring [50]. Further developments in SEM technology may enhance applicability, thus future technological developments should be assessed as they arise [48].

#### Scanning helium microscopy

Helium microscopy (SHeM) presents as an additional emerging, non-destructive microscopy method. This technique utilises the interaction of a ground-state neutral helium atom with a specimen’s surface to produce an image depicting the topographic features and local chemical composition of the surface of a sample without interfering with the sample’s integrity. SHeMs capture an image by propelling a jet of dense helium gas through progressively smaller nozzles until a precise beam of atoms is created. The atoms strike the sample surface, with the backscatter intensity providing information on the local surface’s topography and chemical composition [51].

The theoretical limit for the resolution of images produced through SHeM is the intrinsic wavelength of a helium atom, approximately 0.05 nm, which would provide a level of detail regarding the chemical composition and texture of a target surface that may facilitate image recognition efforts. This theoretical resolution has however yet to be achieved by practical designs [51].

Despite offering high theoretical resolution and valuable local chemical information, SHeM is unlikely to be applied to water quality analysis due to significant image capture time, requirements for the sample to be placed under vacuum, and low spatial resolution available with current technology. Current research efforts aim to optimise helium source designs and develop higher sensitivity detectors to improve image capture rate and spatial resolution. Future developments in SheM should be monitored for viability within a water quality context [51].

## Artificial neural networks

Image recognition by artificial neural networks is a continuously evolving field of research which has recently found a vast array of practical applications within contexts ranging from biometric security to self-driving vehicle guidance. Neural networks can be adapted for cell recognition applications using a range of potential neural network structures, most notably CNNs. Artificial neural network recognition offers an approach to biological data analysis for datasets that are too large for manual analysis, providing a faster and more objective method than human analysis (Shamir et al. 2010). The efficiency of image recognition neural networks can be improved using additional image processing algorithms, including segmentation, focus classification, and deblurring algorithms.

### Post-processing using neural networks

Synergistic use of post-processing and recognition algorithms improves the overall efficiency of the neural networks used, as each component of the network can be optimised to perform a specific task accurately and quickly [47].

#### Image segmentation

Raw image data supplied to recognition algorithms can first be processed by a segmentation neural network to acquire a region of biological significance, while also eliminating optical artefacts such as dust and water droplets. This improves the performance of subsequent recognition algorithms, as fewer irrelevant pixels must be considered and computational resources are instead allocated towards analysing the biological regions of interest [47].

Accurate automatic segmentation of microscopic *Bacillus anthracis* images was achieved by Hoorali et al. [52] using UNet, providing an accurately defined region of biological significance. This technique obviated the requirement for diagnosis by a human specialist, achieving segmentation accuracy of 97% on imaged samples [52]. This segmented image can then be considered by a neural network trained for bacterial recognition, with the risk of the algorithm being misled by optical artefacts or debris mitigated.

For high-throughput imaging methods such as the ultra-large-scale technique developed by Lee et al. [35] segmentation algorithms provide an effective data storage tool, as these algorithms significantly reduce total data storage requirements by eliminating irrelevant background regions. Image segmentation algorithms are thus essential in image post-processing and assists large-scale cyanobacteria image storage and processing efforts.

#### In-focus/out-of-focus classification

Microscopic images captured using flow cytometry typically have shallow depths of focus and feature cells in motion, thus imaged cells are occasionally out-of-focus or subject to motion blur. These images will affect the performance of recognition algorithms and should be filtered out before they are supplied to such an algorithm. Motion-blurred images may be deblurred, while out-of-focus images can be classified and eliminated by algorithms such as DeepFocus. DeepFocus was designed to eliminate out-of-focus tissue cell images to simplify diagnosis efforts, with a similar approach viable for detecting and eliminating out-of-focus cyanobacteria images [53].

#### Deblurring

Many of the microscopy techniques outlined in Table 1 utilise flow cytometry and thus aim to capture high-resolution images of cells in motion, with higher cell throughputs allowing faster sample processing and enhanced data collection rates. Motion blur can however affect the clarity of images captured at higher sheath fluid flowrates, particularly those used in ultrahigh throughput QPI techniques. Generative adversarial networks (GANs) can be used to obtain unblurred (sharp) images from fast-scanned or motion-blurred images, digitally improving the capabilities of the imaging hardware used [54].

Non-blind deblurring uses known algorithms designed for specific optical systems, introducing limitations resulting from changes to imaging conditions and optical configurations [55]. Blind deblurring is more broadly applicable method in which a CNN or GAN directly learns a mapping from a blurred to sharp image. DeblurGAN uses an end-to-end (direct blurred to sharp) deblurring method performed by a trained CNN with novel network architecture to achieve deblurring faster than competing algorithms [54]. Requiring no prior optical system data, the training process involves the estimation of a sharp image from a blurred training image by the CNN, with a GAN simultaneously trained to critique the CNN by considering the difference between the estimated sharp image and the actual sharp image. Over the training process the GAN and CNN are trained in an adversarial manner to improve network performance and image deblurring rates [54]. Use of deblurring algorithms, particularly within high throughput imaging systems, vastly improves the usability of collected data, improving accuracies of enumeration and taxonomic identification efforts.

### Convolutional neural networks

CNNs are a deep feed-forward artificial neural network widely used for image and pattern recognition purposes, offering several advantages when compared with alternative neural networks [56]. Neural networks have been applied widely within biological image recognition applications, including for recognition of bacteria, identification of skin and leaf diseases, cancer cell identification, and tissue classification [20, 57–59]. CNN recognition frameworks are relatively resistant to distortions resulting from differing lighting conditions, optical artefacts, and different image perspectives. CNNs are also less resource intensive to operate as the structure of CNNs reduce the memory requirements of the algorithm. The reduced number of network parameters of within a CNN typically allows a significantly expedited training process relative to that of an alternative neural network with comparable capabilities [56, 59].

#### CNN structure and recognition technique

CNNs are like alternative artificial neural networks in that they emulate biological synapses by firing only when net inputs exceed a certain threshold, i.e. when enough of the content within an examined segment of an image is familiar to trigger a recognition response [20]. Typical CNNs consist of a stacked input layer, an output layer, and several hidden recognition layers within the network. The stacked layers typically consist of convolution layers, non-linear layers, and pooling/subsampling layers [59]. CNNs developed for different purposes utilise different structures appropriate to their purpose.

The convolution layer examines each small block of the provided image to extract useful abstract features [59]. Data provided to the network in the form of images or physical parameters are inputted to the convolution layer expressed as a matrix. This matrix is examined by a smaller filter matrix, called a kernel, which extracts important information from the image to generate a feature map. The stride of the kernel is one of the CNN’s structural parameters, which determines the level of detail extracted from the image. Multiple kernels can be used to yield multiple feature maps, each expressing a different set of sample data [60].

The subsampling layer uses pooling operations on these feature maps, typically min-pooling, max-pooling, and average-pooling, to recognise similar objects in different images. These similarities are manifested in common abstract trends within the feature maps, with statistically similar images categorised by the neural network into a predefined class. For a neural network designed for water quality monitoring, in a multiclass arrangement each class may represent a particular species of cyanobacteria, while in a binary arrangement each class may represent “toxic cyanobacteria” and “non-toxic cyanobacteria”. The performance of recognition algorithms is affected considerably by the class arrangement.

#### Synergistic cell recognition using QPI and CNNs

A particularly promising field of research pertains to the combination of QPI and CNN techniques to identify the cells within a given sample. For a machine learning algorithm that recognises specimens based on learnt examples, identification of cyanobacteria in a sample poses significant difficulty as they exhibit polymorphism, differ greatly in size, and lack taxonomically revealing sexual reproductive structures [18]. As a result, large training sets are required to account for all variations and allow identification of each subspecies.

In the biomedical field, machine learning algorithms have been trained to successfully identify white blood cells and classify them into their subtypes (i.e. B cells and T cells). Researchers determined a 3D refractive index map of the cells examined, providing the neural network with the parameters required for distinguishing between the cell types [32]. While white blood cells are considerably larger than the smaller species of cyanobacteria, a sufficiently high-resolution QPI device will provide a trained CNN sufficient data to identify and distinguish cyanobacteria from other microorganisms and non-biological debris within a given water sample.

In another study, Jo et al. [20] used MATLAB to develop ‘HoloConvNet’, a CNN designed to detect significant biological traits from QPI data to assist with species identification. HoloConvNet was used to identify *Bacillus anthracis* within a dataset containing four additional bacteria strains with similar but differing morphological characteristics, with recognition accuracies up to 96.3% and computational rates of 1 ms/cell achieved by the algorithm [20]. The comparable size of *B. anthracis* and smaller cyanobacteria cells demonstrates the viability and applicability of this analysis technique for water quality monitoring efforts, as the results obtained indicate this technology is sufficiently sensitive for the detection of smaller cells [17, 27].

Researchers also used HoloConvNet to detect and identify *Listeria monocytogenes*, a strain of bacteria previously unseen to the neural network. HoloConvNet successfully identified *L. monocytogenes* without any modification to the algorithm with 85% accuracy as the algorithm automatically detects key biological features for each species presented. This result indicates the versatility of neural networks to identify novel species without significant human intervention. The researchers indicate that a considerably higher accuracy would likely have been achieved if the architecture and learning rules of HoloConvNet were adjusted for identification of this strain [20].

As indicated by the identification accuracy for *L. monocytogenes*, HoloConvNet automatically determines a “cell fingerprint” of important biological traits within images provided to it, allowing direct training of the CNN from raw QPI data. Like other CNNs, HoloConvNet recognises the content of an image by gradually transforming data into a hierarchical representation of the image across several convolution layers, allowing separation into classes [20].

**Fig. 3:**
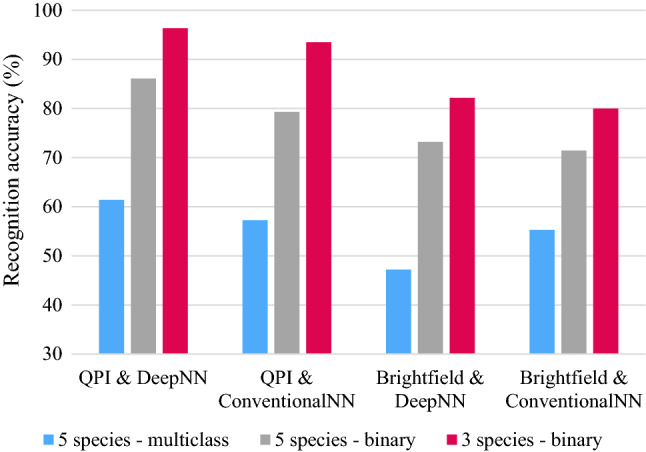
HoloConvNet performance under different testing arrangements. Here “DeepNN” refers to deep neural networks, “ConventionalNN” refers to conventional (single layer) neural networks, “Brightfield” refers to images collected using standard brightfield microscopy. Data [20] adapted from Jo et al.

Figure 3 demonstrates the challenge faced by a CNN trained to recognise and distinguish between individual cyanobacteria species when multiple distinct species are present. HoloConvNet achieved the greatest recognition accuracy when tasked with identifying anthrax in a three species binary-class setup (96.3% accuracy), however when tasked with identifying each specific species in a five species multiclass setup, considerably lower recognition accuracy (approximately 61%) was achieved. These recognition accuracy test results also demonstrate that CNNs achieve the greatest performance when the algorithm is trained to differentiate the data into binary classes (i.e. anthrax or non-anthrax in the case of HoloConvNet). This indicates that greater accuracy can be expected of a cyanobacteria recognition CNN trained to classify specimens within QPI images into such a binary class, such as potentially toxic and established non-toxic species or cyanobacteria and non-cyanobacteria. This approach will provide insight into the health and environmental hazards posed by an examined HAB by quantifying toxic cell presence while minimising the computational resources required.

Figure 3 also reveals the value of the additional information provided by QPI when compared with conventional microscopy. In all testing arrangements, QPI data achieved greater recognition accuracy than conventional brightfield microscopy data. Accuracies 15% higher were achieved using QPI in a three species binary-class test, a trend that was reflected in the five species binary and multiclass tests. These results also indicate that the importance of the imaging method has higher weighting relative to the importance of the neural network used when comparing deep and conventional neural networks [20]. Further research directly comparing the recognition accuracies of AI trained using QPI vs. conventional images should be undertaken to confirm these findings.

#### Training CNNs

The training process of a CNN utilises similar mechanisms and techniques required for the training of other neural networks. Artificial neural networks trained by machine learning attempt to replicate the recognition process occurring within biological neural networks, with the structure of the network consisting of layers of neurons connected by weighted synapses. An activation function regulates the firing strength of the neurons, which may fire when net inputs exceed a certain threshold. The cost function of the algorithm (typically the algorithm’s mean square error) is determined by a comparison of the expected versus actual output of the network, which determines the algorithm’s output error. Backpropagation through the network adjusts the weighting of synapses and allows the iterative improvement of the network through several epochs. The iterative improvements enable learning, which allows the neural network to identify previously unseen images [60, 61].

Training neural networks is a time consuming and computationally intensive process due to the large number of iterations required to form versatile neural pathways [60]. Image recognition neural networks are typically trained through supervised machine learning, an approach in which the algorithm is trained using known examples to learn the distinguishing factors between groups of objects and is then tested on a previously unseen dataset to determine recognition capabilities [47, 61].

While artificial neural network image recognition provides a labour reduction method, the requirement for algorithms to be trained on identified samples requires significant initial labour and expertise to image and identify training specimens. Training set augmentation provides a method for artificially enhancing training set sizes by digitally altering pre-classified images. Identified training images are rotated, flipped, and have noise added through random variation of pixel values [59]. A single data point (e.g. an image of an identified toxic cyanobacteria cell) can be enhanced using this approach to yield over a hundred statistically distinct training examples, which each provide the neural network with an alternate perspective to assist the machine learning process [20, 59].

Despite network architecture improvements relative to other neural networks, the training process for a CNN is still both time and resource intensive. Image recognition neural networks such as those used by researchers to identify bacteria typically take up to two weeks to train on a computer with dedicated graphics processing units (GPUs) [20, 57]. For each trainable category, between 1000 and 10,000 images are required to achieve high accuracy, with 80–85% of these used for training and 15–20% used for testing [57, 61, 62]. Using data augmentation methods described above, the total number of raw images required to train a class can be significantly reduced. Despite this, training a CNN capable of identifying hundreds of distinct cyanobacteria species and determining their viability will pose a significant challenge.

### Methods with emerging viability

#### Capsule neural networks

Capsule Neural Networks (CapsNets) are an emerging network structure offering an alternative mechanism for image recognition to traditional CNNs, with the first successful network developed in 2017 [63]. CapsNet architecture varies from that of a CNN in that they accept and output vectors rather than scalars, allowing CapsNet to learn and account for deformations and altered viewing conditions of the image in addition to learning image features [60]. Each capsule within CapsNet consists of a group of neurons with the output of each neuron representing a different property of the same feature of an object. The network identifies an entire object by first identifying its parts, improving CapsNet’s resilience to alterations of conditions, distortions, and cases in which components of the object are obscured or missing [63]. The ability to recognise a deformed object without additional training may simplify monitoring of water treatment effectiveness by CapsNet, as cell fragments or non-viable cells can be identified and compared against remaining viable cell populations. A CNN trained for this role would require specific training using images of damaged cells and cell fragments. Improved network performance and reduced computational resource reliance relative to CNNs is achieved through a reduction of network parameters, by connecting capsules to other capsules rather than individual neurons connecting to individual neurons (i.e. one capsule to one capsule is one connection, 9 neurons to 9 neurons is up to 9^2^ connections) [60]. Further research aims to determine online trainability of CapsNets, which is not possible in traditional CNNs due to excessive computational resource requirements [60].

## Conclusions from research

A review of machine learning and AI image recognition presented factors that must be prioritised when considering appropriate microscopic imaging techniques. These factors included:

1. *The rate and ease of image capture* neural networks require large and diverse training sets to allow accurate recognition of a certain class. As discussed previously, multiple classes may exist for each species of cyanobacteria due to polymorphism and investigations of cell fragments during viability analysis. Resultantly, many images must be captured of each class in every possible configuration (e.g. images of different cell orientations, cell fragments, etc.). The microscopic imaging method must therefore allow a rapid sample throughput.
2. *The information provided by captured images* Enhanced information availability provides a detailed “cell fingerprint” and improves the ability for a neural network to identify classes. Information can be enhanced through improved spatial resolution, imaging in three dimensions, or by using an imaging method that captures additional biophysical cell parameters. For a target class that lacks an abundance of distinguishing physical characteristics (such as bacteria) the availability of additional biophysical cell information is of crucial importance

After considering the range of microscopic imaging methods used within the scientific literature and the capabilities of commercially available equipment, quantitative phase imaging was identified as the most viable approach. Considering the above factors, QPI equipment can be readily integrated with flow cytometry to allow rapid sample processing, and the enhanced information availability allows for greater identification accuracy when compared to brightfield microscopy images.

## Recommendations

**Fig. 4:**
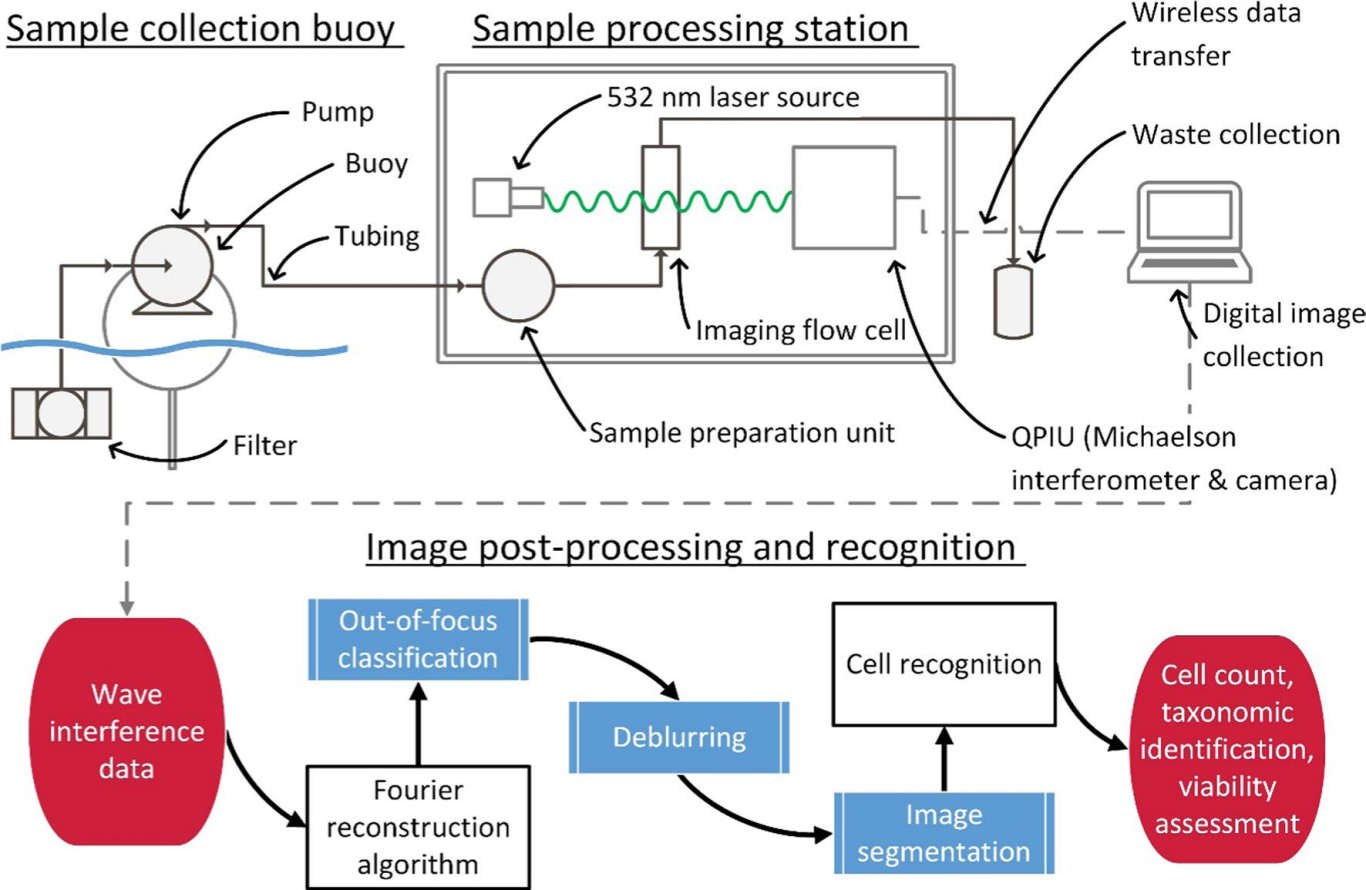
Cyanobacteria monitoring workflow from sample collection to image processing

This section further outlines and considers techniques discussed within this review to propose an optimised design of a water quality monitoring system. Consideration of the reviewed microscopic imaging methods and the reviewed training requirements, strengths, and weaknesses of AI image recognition, quantitative phase imaging presents the most promising potential technique. This section outlines the advantages and disadvantages of three approaches to QPI successfully used within the literature for cell imaging. The methods are then compared, revealing Method 2 (QPI using Michelson interferometry) as the most viable technique. This allowed the construction of a preliminary design for a water quality monitoring station (Fig. 4).

### Method 1: portable quantitative phase imaging unit

A small device (discussed in section “Portable QPIU”) attached to the output of a standard brightfield microscope (designated Method 1 hereafter) is considered for its potential to assist recognition of cells by a trained convolutional neural network. Viability of this technique is indicated by the recognition accuracies approaching 95% achieved by the previously discussed trained CNN “HoloConvNet” [20].

#### Advantages

The primary advantage of Method 1 lies in the simplicity and portability of the design, offering a valuable potential as a proof-of-concept design. The image collection method is simple and optical calibration requires the consideration of few components. The assembly process for this device is also simple and does not require disassembly between data recording instances, so can be easily transported between locations.

As previously discussed, data provided by Method 1 has been successfully used for bacteria recognition by a trained CNN. Following appropriate adjustments of algorithm parameters, bacteria imaged using this method were recognised with accuracies up to 96.3%. The same QPIU and CNN was also used to image and successfully identify *L. monocytogenes* without any modification to the algorithm with 85% accuracy, indicating the versatility of this design.

#### Disadvantages

The primary disadvantage of Method 1 is the manual sample analysis and preparation process. As discussed previously samples must be manually placed within an imaging chamber in the space between two microscope slides. The microscope must be manually focussed and must be immersed in oil to achieve high spatial resolution (discussed below). The manual sample preparation and analysis technique will considerably reduce sample throughput rates relative to automated techniques such as Method 2, which captures 100 images/second of cells in flow. Manual sample preparation also requires technical expertise from the microscope operator, introducing unnecessary labour costs and training requirements [15]. This method has the potential for integration with flow cytometry equipment by substituting the imaging chamber with an imaging flow cell; however, this would require a redesign of the experimental setup and require additional equipment purchases.

Method 1 provides a portable device for capturing QPI images, however the portability is limited as a brightfield microscope in a laboratory context is required for maximum magnification. As such, portability of this device between laboratories is simple; however, the in situ applications are very limited.

This method provides images with high (comparable with Method 2) spatial resolution; however, this resolution relies on an oil immersion technique to achieve 100X objective magnification. This complicates the imaging process as each individual slide containing a new sample must have oil manually applied prior to imaging.

### Method 2: QPI using Michelson interferometry

A QPIU using Michelson interferometry (discussed in section “Michelson interferometry”), Method 2 hereafter, provides detailed analysis of biophysical cellular features including radius and dry mass utilising phase-shifting quantitative phase imaging. Automated sample processing and data collection using integrated flow cytometry enhances data collection rates [37]. The advantages and disadvantages of this method are further considered within this section.

#### Advantages

The primary advantage of Method 2 is the high spatial resolution attainable in images captured using this method, providing precise information on biophysical cellular properties. Unlike Method 1, this accuracy does not rely on oil immersion, which removes a preparation step and simplifies the imaging preparation process. The viability this method was demonstrated through the accurate images captured of tumour cells with diameters of 7–9 μm, which is comparable to the sizes of smaller cyanobacteria species [17]. If examined cyanobacteria species are too small to be accurately imaged with this method, the modular design allows for components such as the microscope objective to be substituted with higher-magnification equivalents.

Another significant advantage of Method 2 is the automated sample analysis method provided through integration with flow cytometry equipment to significantly improve sample processing rates, allowing accelerated data collection relative to manual analysis techniques [15].

Considering the costs for the three methods outlined in this section, Method 2 provides the most economical design. The cost is comparable to that of Method 1, but significantly cheaper than that of Method 3.

#### Disadvantages

While the interferometry design allows a precise interference pattern to be created, this also poses as a disadvantage of Method 2. The interferometer requires precise calibration to achieve an interference pattern on the scale of tens of nanometres. The considerable number of components also introduces difficulty in troubleshooting potential issues, as there are more possible fail points in the design.

Method 2 suffers from the lowest portability of the three methods considered, which is an additional symptom of the complexity of the design. Any transportation of the interferometer will require disassembly and recalibration upon reassembly, with vehicular transportation preferable. The components used in this design are sensitive and thus must be protected from environmental affects to preserve precision and calibration. While a laboratory is not necessarily required, somewhat stable conditions (e.g. within a weatherproofed shed) are required.

### Method 3: QPI using 4Deep S7 submersible microscope

Method 3 is an early example of commercially available QPI technology, manufactured by 4Deep. The device (discussed in section “4Deep S7 submersible microscope) is a small, portable probe designed for in situ analyses, and has been used to image microscopic marine life in the high Arctic [41].

#### Advantages

Method 3 is a small device designed for in situ analyses, offering significant flexibility when compared with Method 1–2. Since it is designed for oceanic applications in which considerable temperature and pressure variations are anticipated, the conditions experienced within a lake are unlikely to pose significant operational issues. The small physical size of the device simplifies transportation, allowing rapid relocation between testing instances.

Method 3 is a commercially available product, with manufacturer support and software offered along the physical product. Method 3 has inbuilt quantitative phase reconstruction capabilities, eliminating a step of the potential image processing method outlined in Fig. 2. Manufacturer support also simplifies the equipment preparation stage, since the optics will have already been calibrated and optimised by the manufacturer.

#### Disadvantages

The fundamental disadvantage of Method 3 is the design of device as a passive probe, complicating quantitative analyses. A similarly designed device utilising a small-scale flow cytometer would allow accurate cell counts; however, at the time of writing such a device is not commercially available.

Relative to Methods 1–2, the considerable price of Method 3 poses a significant disadvantage for prospective end users. Since an ideal cyanobacteria monitoring setup will utilise multiple instruments at multiple locations of a body of water, the improved image information provided by such an expensive device relative to standard fluorescence monitoring is difficult to justify.

Another disadvantage of Method 3 is the low spatial resolution of images captured using this device relative to Methods 1–2. The manufacturers indicate that specimens no smaller than 20 µm can be accurately imaged, which prohibits the analysis of many smaller cyanobacteria species and resultantly limits the value of Method 3 as a water quality microscopic imaging technique [17].

### QPI comparison

Table 3 outlines a comparison of the performance of three QPI methods discussed in this section with respect to important attributes. Considering the attributes outlined above, Method 2 presents the greatest ability for workflow optimisation. The low portability of this method is unlikely to pose a significant disadvantage if the device is assembled within a fixed monitoring location (Fig. 4). The disadvantages posed by such an arrangement are considered in below with potential mitigation measures discussed. Excluding the previously mentioned low portability, Method 2 performs at an equivalent or superior level to the alternative methods outlined within Table 3.

**Table 3:** QPI unit comparison, colour coded for simplified visual analysis

Attribute	Method 1 (portable QPIU)	Method 2 (QPI using Michelson interferometry)	Method 3 (4Deep S6 submersible microscope)	
Portability	High	Low		High
In situ applications (without additional equipment)	Low	Low		High
Sample processing/preparation	Manual	Automatic		N/A
Spatial resolution	High (relies on oil immersion)	High		Low
Cost	Low	Low		High
Commercial availability	N/A	N/A		Yes
AI image recognition	Proven (HoloConvNet)	Unproven but applicable		Insufficient for smaller (< 20 µm) cells

### Experimental design

Figure 4 outlines a proposed implementation of Method 2 (QPIU using Michelson interferometry) to an in situ monitoring location. The QPIU is assembled within a small weatherproof structure such as a shed located adjacent to a monitored body of water, or within a water treatment plant. Sample collection buoys are situated across the body of water in desired regions and connected by tubing to the processing station, using a small pump mounted on the buoy to transfer sample solutions. This tubing must be periodically flushed out or replaced to prevent build-up of cyanobacteria/algal biomass, which may affect the accuracy of cell counts and taxonomic identifications. Tubing can be submerged in waterways with high vehicular traffic, preventing disruptions and mitigating risks of the tube sustaining damage. Using a sample preparation unit such as the OC-300 (discussed in section “Sample Preparation – OC-300”), up to 12 parallel streams can be processed from 12 separate sample collection buoys. The sample buoys have a length of tubing extended a desired depth into the water, at the end of which a filter mesh is positioned to allow cyanobacteria to enter the tubing but prevent debris such as plant matter and small animals from entering. This mesh will also require periodic maintenance to prevent blockages.

Initial implementations will require manual relocation of sample buoys if monitoring of alternate locations is desired. Future improvements may involve a renewably powered GPS-linked buoy with a small electric motor, allowing automatic remote-controlled relocation to different regions of interest.

The water samples are then processed with 100 images per second captured of any specimens or debris passing through the imaging flow cell using Method 2. Flow cytometry performance can be improved by programming the OC-300 to introduce Lugol’s solution during the sample preparation phase, which will reduce adhesion between cells in the sheath fluid.

A Fourier reconstruction algorithm is then used to retrieve quantitative phase images from the interference pattern captured by the CMOs camera. The reconstructed QPI images are then provided to a series of post-processing neural networks (discussed in “Post-processing using neural networks”) to improve the overall performance of the network. These networks include an out-of-focus classifier, a deblurring algorithm, and an image segmentation algorithm. A neural network trained to identify cyanobacteria in QPI images then analyses approximately 1 cell/millisecond, delivering a quantitative summary of the taxonomic composition of the sample and determines a cell count per millilitre using the precise volume of the sample prepared by the OC-300. For measurements conducted in treatment plants, the neural network can be trained to determine cell viability and the portion of cells destroyed by treatment process. The number of out-of-focus images, as well as the number of unidentified objects in the sample, are then used to determine an uncertainty interval for the cell count and sample composition. Unidentified cells are flagged by the network for inspection and identification by an operator, from which the neural network can undergo further training. Initially a neural network developed using MATLAB can provide limited recognition capabilities. The post-processing and recognition process will likely require a dedicated computer to facilitate continuous sample processing from several sample collection buoys.

This data can be summarised and used to provide additional training samples for further AI development, evaluate the efficacy of water treatment processes used, and be provided to a risk assessment team to assists tailored water quality risk assessments considering cyanobacteria taxonomy and cell counts.

### Recommendation for further research

The proposal outlined above represents only a preliminary design. A practical implementation will require further research and consideration of many additional factors and design parameters. Several key considerations for further research have been outlined below:

- Determination of pertinent interferometer design information (e.g. distances between components, lens angles, total desired magnification, etc.)
- Compatibility of optics components
- Optimal imaging conditions (e.g. external lighting)
- Field retrieval method used to reconstruct QPI images (e.g. Fourier transforms and numerical propagation by angular spectrum method)
- Optimal segmentation algorithm
- Optimal refocussing/deblurring algorithm
- Optimal CNN platform and parameters for image interpretation
- Investigation of the performance of image recognition for 2D vs 3D QPI images (2D is less computationally intensive but 3D offers enhanced information)
- Cost optimisation

QPI is the subject of continued research and development. Method 3 highlights the growing commercial interest in this technology. Increasing prevalence of machine learning as a means of processing large data sets is likely to foster further development. A future design comparable to the in situ (4Deep S7 Submersible Microscope) device but utilising integrated flow cytometry capabilities and improved spatial resolution will provide a sophisticated platform from which cyanobacteria populations can be monitored in real time.

## Conclusion

Technologies and techniques within the published literature were reviewed to propose a real-time cyanobacteria monitoring process using quantitative phase imaging microscopy and machine learning neural network cell recognition. Real-time microscopic monitoring and evaluation of microbial activity allows early HAB detection, water treatment efficacy evaluation, and informs accurate risk assessments by considering taxonomy and cell counts. Three potential QPI techniques (Method 1–3) were evaluated, the most viable of which were used to propose an optimised cyanobacteria monitoring station (Fig. 4). The recommendations within this report thus provide a theoretical basis from which a practical water quality monitoring system can be constructed to simplify and improve HAB and cyanobacteria monitoring.
